# The Effect of FGF21 and Its Genetic Variants on Food and Drug Cravings, Adipokines and Metabolic Traits

**DOI:** 10.3390/biomedicines9040345

**Published:** 2021-03-29

**Authors:** Sarah Epperlein, Claudia Gebhardt, Kerstin Rohde, Rima Chakaroun, Marie Patt, Imke Schamarek, Susan Kralisch, John T. Heiker, Markus Scholz, Michael Stumvoll, Peter Kovacs, Jana Breitfeld, Anke Tönjes

**Affiliations:** 1 Medical Department III–Endocrinology, Nephrology, Rheumatology, University of Leipzig Medical Center, 04103 Leipzig, Germany; sarah.epperlein@medizin.uni-leipzig.de (S.E.); rima.chakaroun@medizin.uni-leipzig.de (R.C.); marie.patt@medizin.uni-leipzig.de (M.P.); imke.schamarek@medizin.uni-leipzig.de (I.S.); susan.kralisch@medizin.uni-leipzig.de (S.K.); michael.stumvoll@medizin.uni-leipzig.de (M.S.); peter.kovacs@medizin.uni-leipzig.de (P.K.); 2 Helmholtz Institute for Metabolic, Obesity and Vascular Research (HI-MAG) Helmholtz Center Munich at the University of Leipzig and the University of Leipzig Medical Center, 04103 Leipzig, Germany; claudia.gebhardt@helmholtz-muenchen.de (C.G.); kerstin.rohde@helmholtz-muenchen.de (K.R.); john.heiker@helmholtz-muenchen.de (J.T.H.); 3 Institute for Medical Informatics, Statistics and Epidemiology, Medical Faculty, University of Leipzig, 04107 Leipzig, Germany; markus.scholz@imise.uni-leipzig.de; 4 Deutsches Zentrum für Diabetesforschung, 85764 Neuherberg, Germany

**Keywords:** fibroblast growth factor 21, eating behavior, addictive behavior, adipokines, genetic variation in *FGF21*

## Abstract

Fibroblast growth factor 21 (FGF21) is a regulator of addictive behavior. Increasing evidence suggests an impact of FGF21 on eating behavior, food and drug cravings and on other adipokines like insulin-like growth factor 1 (IGF-1) or adiponectin. We investigated the association of serum FGF21 and genetic variants with aspects of food and drug craving and obesity related metabolic parameters including serum adipokine levels. Standardized questionnaires, blood samples and anthropometric data of the Sorbs cohort (*n* = 1046) were analyzed using SPSS. For genetic analyses, the *FGF21*-locus ±10 kb was genotyped and analyzed using PLINK. Validation was conducted in a second independent cohort (*n* = 704). FGF21 was significantly associated with alcohol and coffee consumption, smoking and eating behavior (disinhibition). We confirmed correlations of FGF21 serum levels with IGF-1, adiponectin, pro-enkephalin, adipocyte fatty-acid-binding protein, chemerin and progranulin. *FGF21* genetic variants were associated with anthropometric and metabolic parameters, adipokines, food and drug craving while strongest evidence was seen with low-density lipoprotein cholesterol (LDL-C). We highlight the potential role of FGF21 in food and drug cravings and provide new insights regarding the link of FGF21 with other adipokines as well as with metabolic traits, in particular those related to lipid metabolism (LDL-C).

## 1. Introduction

The human fibroblast growth factor 21 (FGF21) is a protein consisting of 209 amino acids [1] which is encoded by the *FGF21* gene on chromosome 19q13.33 [2]. In humans, *FGF21* is mainly expressed in the liver, but also to a minor extent in the thyroid gland [2]. An increase of circulating FGF21 can be observed e.g., after acute exercising [3], long-term fasting [4], very low caloric diet [5], excessive intake of carbohydrates [6], in the postprandial period [7], after alcohol consumption [8], and lipid infusion [9], in type 2 diabetes (T2D) [10], metabolic syndrome (MS) and obesity [11,12]. Several studies in mice showed FGF21 to suppress ad libitum alcohol intake [8], to have a glucose and lipid lowering effect [13] as well as an insulin sensitizing [14] effect. Furthermore, FGF21 is thought to induce weight loss in obese mice [15], probably through affecting peripheral tissues, for example by upregulating glucose transporter 1 (GLUT 1) in adipocytes and by increasing peripheral glucose expenditure [16]. In humans, FGF21 regulates glucose uptake in monocytes in vitro, may have an impact on immune response [17] and correlates with impaired glucose tolerance, serving as a predictor for T2D [12,18,19]. Moreover, FGF21 as a potential biomarker of non-alcoholic-fatty-liver disease (NAFLD) may play a role in hepatic lipid metabolism as its serum concentration is elevated in NAFLD and its expression correlates positively with liver steatosis [20]. Furthermore, the relationship of FGF21 with other adipokines has recently been addressed. Exemplarily, FGF21 inhibits insulin-like growth factor 1 (IGF-1) secretion in vitro [21] and its activity [22]. In Chinese children with obesity, high FGF21 serum levels correlated inversely with adiponectin levels implicating a potential FGF21 resistance in obese states [23], since FGF21 treatment of mice results in increased adiponectin secretion [24]. Finally, studies in mice also demonstrated that FGF21 signals to the central nervous system e.g., via the paraventricular nucleus to suppress food intake [25]. Moreover, experimental models suggested a potential role of FGF21 in addictive behavior by showing that suppression of alcohol and sweets consumption by FGF21 administration is accompanied by a lower concentration of dopamine in the central nervous system [26].

Based on the current knowledge, FGF21 has become a promising target for pharmacotherapy. Indeed, FGF21-analogons administered to patients with obesity and T2D resulted in weight loss, lower low-density lipoprotein cholesterol (LDL-C) and triglycerides (TG), higher high-density lipoprotein cholesterol (HDL-C), but also higher blood pressure. Additionally it improved fasting insulin (FI) and adiponectin levels [27,28,29]. Subcutaneous administration of a FGF21/β-klotho complex reduced body weight and the cardio-metabolic risk in humans while data also suggest effects on sweet preference and the consumption of carbohydrate rich foods [30].

In line with the physiological effects of FGF21, genetic variants within or near the *FGF21* locus were shown to be associated with changes in eating behavior, consumption of addictive substances and metabolic and anthropometric parameters [31,32,33,34,35]. However, in the majority of the studies, circulating FGF21 levels were not measured and therefore prediction of potential causal mechanisms was not possible [31,33,34]. Thus, one objective of this study was to investigate the association of FGF21 serum concentrations with food and drug craving (eating behavior, alcohol, coffee, smoking). Given the proposed relationship of FGF21 with other adipokines, we tested the association of FGF21 with numerous adipokines, namely chemerin, vaspin, pro-neurotensin (pro-NT), pro-enkephalin (PENK), progranulin, adiponectin, adipocyte fatty-acid-binding protein (AFABP), irisin, angiopoietin-related growth factor (AGF), and IGF-1. Finally, we examined whether common genetic variants in the *FGF21* locus were related to circulating FGF21 serum levels, the expression of *FGF21* in peripheral blood mononuclear cells (PBMCs), metabolic and anthropometric phenotypes including the measured adipokines and food and drug craving.

## 2. Materials and Methods

### 2.1. Subjects

Participants included in the present analyses were part of the Sorbs cohort from Eastern Germany (described in detail elsewhere [21,36,37,38]). Briefly, the Sorbs cohort contains a total of 1046 subjects. Regarding clinical association analyses 780 subjects (330 males and 450 females) were included for which serum FGF21 level were available. For genetic association analyses the entire cohort of *n* = 1046 was included. Baseline characteristics of the study subjects are shown in Table 1. The study has been approved by the ethic committee of the University of Leipzig (Reg. No.: 088-2005 and for the adipokine genome-wide association study A.Z.: 330-12-24092012).

To validate findings concerning FGF21 and alcohol consumption or smoking, we analyzed these traits in a separate validation cohort from Leipzig (Table 2). This cohort consisted of a subgroup (*n* = 704) of larger recruitment efforts at the Integrated Research and Treatment Center for Adiposity Diseases (Leipzig, Germany) to assess metabolic disease progression in a wide spectrum of phenotypes, ranging from healthy controls, metabolic disease, lean and overweight subjects with T2D and subjects with morbid obesity. Subjects were screened for standardized inclusion and exclusion criteria. The latter comprised acute or chronic inflammatory or infectious diseases, history of organ transplantation including immunosuppressive treatment, severe kidney failure (estimated glomerular filtration rate (eGFR) 50 mL/(min·1.73 m^2^)) and drug or alcohol addiction.

### 2.2. Phenotyping

#### 2.2.1. Sorbs Cohort

Anthropometric data and data of past medical history (smoking, alcohol and coffee consumption) were collected using standardized questionnaires. Body impedance analyses and a 75-g oral glucose tolerance test (OGTT) was performed and insulin resistance was defined by calculating the homeostasis model assessment for insulin resistance (HOMA-IR) as described elsewhere [39].

Blood samples were drawn in the morning after overnight fasting. By using standard laboratory methods LDL-C, HDL-C, TG, γ-glutamyltransferase (γGT), alanine aminotransferase (ALAT), aspartate aminotransferase (ASAT), alkaline phosphatase (AP), fasting glucose (FG), hemoglobin A1c (HbA1c (%)) were determined in a certified laboratory (University of Leipzig Institute of Laboratory Medicine). Serum insulin levels were measured by the AutoDELFIA Insulin assay (PerkinElmer Life and Analytical Sciences, Turku, Finland). Serum concentrations of FGF21 and other adipokines (chemerin, vaspin, pro-NT, PENK, progranulin, adiponectin, AFABP, irisin, AGF, IGF-1) were determined using commercially available enzyme-linked immunosorbent assays (ELISAs) following the manufacturer’s protocol. The limit of detection for serum FGF21 concentrations was 7 ng/L, the intra-assay was 2% and inter-assay 3.3% (BioVendor, Brno, Czech Republic). Quality criteria and characteristics of the laboratory methods, the AutoDELFIA and the applied ELISAs were described in detail recently [21,40]. The mRNA expression of *FGF21* was assessed in PBMCs extracted from anti-coagulated blood samples [41] and DNA was extracted and genotyped as described in detail elsewhere [36,37,38]. Briefly, the QIAmp DNA Blood Midi Kit (Qiagen Inc., Valencia, CA, USA) was used to extract genomic DNA following the manufacturer’s instructions and genotyping was performed using the 500K Affymetrix GeneChip and the Affymetrix Genome-Wide Human SNP Array 6.0 (Affymetrix Inc., Santa Clara, CA, USA) by the Microarray Core Facility of the Interdisciplinary Center for Clinical Research (University of Leipzig, Germany) and by ATLAS Bio-labs GmbH (Berlin, Germany). The genotypes have further been determined using the GeneChip Genotyping Analysis Software (GTYPE) and the BRLMM algorithm for the 500K arrays and the Birdseed Algorithm for Genome-Wide Human SNP Array 6.0 (Affymetrix Inc.). For gene expression in PBMCs the blood samples have been collected in VACUTAINER CPTs (Cell Preparation Tubes) containing sodium heparin as anti-coagulant. To extract the PBMCs the manufacturer’s protocol was used (BD, Franklin Lakes, NJ, USA). Further, RNA was extracted using the TRIzol protocol (Thermo Fisher Scientific), followed by DNase I digestion and clean-up using the RNeasy MinElute Cleanup Kit (Qiagen, Hilden, Germany). Illumina GeneChip analyses was done at the microarray core facility of the Interdisciplinary Centre for Clinical Research (ICCR) in Leipzig (Dr. Krohn, Faculty of Medicine, University of Leipzig) and the Illumina Human HT-12 v4 Bead Chip was used according to the manufacturer’s instructions.

Eating behavior was assessed by the German Version of the Three-factor eating questionnaire, referred to as “Fragebogen zum Essverhalten (FEV). The FEV assesses three domains of eating behavior: “disinhibition” is comprised of 16 items and is defined as being unable to control food intake under various circumstances for example due to emotional stress. “Hunger” comprises 14 items and is defined as intense sensing of hunger signs. The domain “restraint” comprised of 21 items, describes the ability of cognitive control of eating behavior to reduce or maintain body weight. Higher scores within a single domain indicate that an individual’s eating behavior is best described by this domain [42,43,44,45].

#### 2.2.2. Validation Cohort from Leipzig

Phenotyping of the validation cohort was performed according to standard operating procedures including the assessment of clinical parameters and anthropometric data as well as the acquisition of biological samples. Dietary assessment was performed using a validated semiquantitative Food Frequency Questionnaire (FFQ) [46]. The layout and format were based on the European Prospective Investigation of Cancer (EPIC)-Norfolk FFQ. Portion size and nutrient composition were derived from national food consumption surveys and food composition databases as previously described [46]. Smoking status was assessed in a dichotomous manner evaluating current active/passive status (yes/no). Whole blood was spun down for serum extraction and serum samples were stored at −80 °C until further processing. FGF21 was measured in serum samples using an ELISA sandwich assay according to manufacturer’s recommendations (BioVendor) in 492 subjects.

### 2.3. Statistical Association Analyses

#### 2.3.1. Clinical Parameters

In the Sorbs cohort, FGF21 serum measurements were performed for 927 subjects. 147 participants displayed serum concentration below the detection limit. Thus, FGF21 serum levels were available in 780 subjects. Clinical association analyses of serum FGF21 levels were performed using SPSS (IBM Corp. Released 2016. IBM SPSS Statistics for Windows, Version 24.0. IBM Corp., Armonk, NY, USA). Multiple linear regression analyses were performed separately for each dependent variable: disinhibition score, hunger score, restraint score, pack years (number of packs of cigarettes/day × amounts of active smoker years) and *FGF21* mRNA expression in PBMCs. For ordinal-scaled parameters (alcohol and coffee) an ordinal regression (generalized linear model) was performed. These traits were categorized as followed: alcohol (1 = no consumption, 2 = occasionally/at parties, 3 = two to three glasses of wine or bottles of beer/week, 4 = one glass of wine or one bottle of beer/day, 5 = two glasses of wine or bottles of beer/day, 6 = three or more/day); coffee (0 = no consumption, 1 = one cup/day, 2 = two cups/day, 3 = three cups/day, 4 = four or more cups/day). To test whether serum FGF21 concentrations were associated with smoking status, binary logistic regression was applied. To analyze the relationships of different adipokines and FGF21, Spearman’s rank correlation coefficient was calculated and partial correlation including adjustments for age, sex and body mass index (BMI) was conducted. All continuous parameters (serum FGF21 concentrations, age, BMI, adipokines, pack years and expression parameters) were ln-transformed before entering statistical analysis.

In the validation cohort, analyses were performed using SPSS. Continuous parameter (serum FGF21 concentrations, age, BMI, alcohol consumption) were ln-transformed before entering statistical analysis. The association between alcohol consumption and FGF21 serum concentrations was investigated using linear regression analysis as the applied score of ‘food alcohol’ is a continuous parameter in contrast to the ‘score alcohol’ in the Sorbs cohort which is ordinal scaled. To test whether FGF21 concentrations significantly differed between smokers and non-smokers binary logistic regression was performed.

#### 2.3.2. Genetic Association Studies

We performed genetic association analyses using PLINK in the Sorbs [47]. With the exception of analysis including serum FGF21 concentrations (*n* = 780), all subjects were included (*n* = 1046). The analysis of parameters of lipid metabolism (TG, LDL-C, HDL-C) included only subjects without lipid-lowering medication (*n* = 678). 15 single nucleotide polymorphisms (SNPs) from the *FGF21* gene locus ±10 kb filtered from the genome wide data set using imputed variants based on 1000 Genome information [48] were analyzed. A brief description of the genetic variants is provided in the Supplementary File 1. Inclusion thresholds were set as followed: minor allele frequency 0.01, genotyping rate 0.9 and Hardy-Weinberg-Equilibrium 0.0001. One SNP (rs499765) failed the Hardy-Weinberg test and was excluded from further analyses. For the remaining 14 SNPs linear regression analyses were performed for all traits except for alcohol, coffee and smoking (yes/no) which were analyzed using logistic regression models. Ordinal traits were transformed to binary coded variables before being taken forward to logistic regression analyses using PLINK. Smoking was coded to 1 = no smoker and 2 = smoker; alcohol consumption was coded to 1 = low consumption (scores 1 to 3 from the scales above) and 2 = high consumption (scores 4 to 6 from the scales above); coffee consumptions was coded: 1 = low consumption (scores 0 to 1 from the scales above) and 2 = high consumption (scores 2 to 4 from the scales above). All analyses were adjusted for sex, age and BMI (for analyses of BMI only age and sex were considered). All variables were ln-transformed except alcohol, coffee, scores of eating behavior and sex. Results were condensed into 8 linkage disequilibrium (LD-) groups (LD-*R*^2^ 0.8) showing values of the representative SNP which we chose to be the one with the most associations to different traits (Table 3), results of all analyzed SNPs are shown in Supplementary Files 2–7.

## 3. Results

### 3.1. Association of Serum FGF21 Concentrations with Clinical Traits

#### 3.1.1. Food and Drug Cravings

In the Sorbs, serum FGF21 concentrations were associated with disinhibition (β = −0.152, *p* = 0.001), which remained significant even after adjustment for sex, age and BMI (β = −0.158, *p*_adj_ = 2.17 × 10^−4^). Serum FGF21 concentrations significantly associated with increased risk of being a smoker (odds ratio (OR) (95% confidence interval) = 1.34 (1.16; 1.55), *p* = 7.20 × 10^−5^ and OR_adj_ (95% confidence interval) = 1.39 (1.18; 1.64), *p*_adj_ = 6.70 × 10^−5^) and a more intense consumption of alcohol (OR = 1.32 (1.16; 1.49), *p* = 1.30 × 10^−5^ and OR_adj_ = 1.28 (1.13; 1.46), *p*_adj_ = 1.51 × 10^−4^). In line with this, elevated circulating FGF21 level were associated with increased pack years in smokers (β = 0.239, *p* = 6.80 × 10^−5^ and β_adj_ = 0.111, *p*_adj_ = 0.030). In contrast FGF21 concentrations were associated with a lower intense of coffee consumption (OR = 0.87 (0.77; 0.99), *p* = 0.029 and OR_adj_ = 0.80 (0.70; 0.91), *p*_adj_ = 0.001) (Table 4).

We also found a significant association between serum FGF21 concentrations and alcohol consumption in our validation cohort (food alcohol: β = 0.162, *p* = 3.00 × 10^−4^, and β_adj_ = 0.164, *p*_adj_ = 1.00 × 10^−3^) (Table 5). In line with our findings in the Sorbs, higher FGF21 serum concentrations also associated with increased risk of being identified as smoker in the validation cohort (OR = 1.32 (1.07; 1.64), *p* = 0.011 and OR_adj_ = 1.67 (1.33; 2.16), *p*_adj_ = 4.10 × 10^−5^) (Table 5).

Beyond that, the association of mRNA expression of *FGF21* in PBMCs and serum FGF21 concentrations was significant (β = 0.083, *p* = 0.022) and independent of age, sex and BMI (*p* 0.05 for each single variable; β = 0.069, *p* = 0.067 adjusted for all three parameters).

#### 3.1.2. Adipokines

Among the analyzed adipokines, AFABP, chemerin and IGF-1 showed strongest significant partial correlations with serum FGF21 concentrations (all *p* 0.001 adjusted for age, sex, BMI): AFABP (β = 0.248), chemerin (β = 0.200) and IGF-1 (β = −0.195). Further, significant correlations of serum FGF21 comprised progranulin (β = 0.098, *p* = 0.019) and PENK (β = 0.092, *p* = 0.028), whereas adiponectin, irisin, vaspin, AGF and pro-NT were not significantly correlated with circulating FGF21 concentrations including the adjustments. However, in Spearman’s rank correlation without adjustment adiponectin showed significant correlation with FGF21 (*p* = −0.092, *p* = 0.013) (Table 6).

### 3.2. Association of Genetic Variants in FGF21 with Metabolic Traits

#### 3.2.1. Association of rs838133 with FGF21 Concentrations and Metabolic Traits Related to Obesity

After quality control 14 common variants in the *FGF21* locus ±10 kb within eight different LD-groups were selected for further analyses (Table 3). We performed association analyses with the recently described SNP rs838133, whose A-allele has been shown to be associated with changes in food and drug craving and metabolic and anthropometric parameters [31,32,49,50]. In the present study the minor G-allele was significantly associated with lower LDL-C in the Sorbs cohort (β = −0.034, *p* = 0.005); (LD2 in Table 7, Supplementary File 2).

#### 3.2.2. Effects of Common Genetic Variation within the FGF21 Locus ± 10 kb

Three SNPs from two different LD-groups were significantly associated with serum FGF21 level (Table 8): the SNPs in LD-group 1—rs4015 (β = 0.227, *p* = 0.010) and in LD-group 4—rs11667321, rs8106205 (β = 0.108, *p* = 0.037) (for details see Supplementary File 3). Two independent SNPs were significantly associated with height: rs4021 (β = −0.004, *p* = 0.031, LD-group 3) and rs838143 (β = −0.009, *p* = 0.003, LD-group 7). The same two independent SNPs were significantly associated with weight: rs4021 (β = −0.009, *p* = 0.022, LD-group 3) and rs838143 (β = −0.018, *p* = 0.003, LD-group 7) (in detail see Supplementary File 4).

Two LD-groups showed a significant association with FI (Table 7): rs4015 (β = 0.128, *p* = 0.002) and rs12611028 (β = 0.120, *p* = 0.008) from LD-group 1 and rs3826821 (β = 0.111, *p* = 0.033) from LD-group 6. Furthermore, two of these SNPs were significantly associated with HOMA-IR: rs3826821 (β = 0.135, *p* = 0.017, LD-group 6) and rs4015 (β = 0.109, *p* = 0.019; LD-group 1) (Table 7; details provided in Supplementary File 5). Nine SNPs from four different LD-groups showed a significant association with LDL-C: LD-group 2: rs838133 (β = −0.034, *p* = 0.005) and rs838144 (β = −0.035, *p* = 0.003); LD-group 4: rs12975033, rs8104897, rs8105137, rs8106205, rs11667321 (all *p* 0.001); LD-group 5 with rs12975781 (β = −0.032; *p* = 0.018); and LD-group 8 with rs838136 (β = −0.041, *p* = 0.005) (Table 7; see Supplementary File 2). In the analyses of liver parameters, we observed a significant association of rs838143 (LD-group 7) with γGT (β = −0.113, *p* = 0.034) and of rs12975033 (LD-group 4) with ASAT (β = −0.022, *p* = 0.047) (Table 7; for details see Supplementary File 6). In addition to the association with LDL-C, the LD-group 4 variants (with rs12975033, rs8104897, rs8105137) showed a significant effect on IGF-1 (all *p* 0.037) (Table 9; see Supplementary File 3). Further associations with adipokines were seen for rs4015 (LD-group 1) which was significantly associated with adiponectin (β = 0.057, *p* = 0.031) and with AFABP (β = 0.088, *p* = 0.028), for rs4021 (LD-group 3) which was significantly associated with pro-NT (β = −0.044, *p* = 0.045), and rs3826821 (LD-group 6) which was significantly associated with PENK (β = 0.066, *p* = 0.009) (Table 9; Supplementary File 3).

In the category of food and drug craving (Table 10; details provided in Supplementary File 7) rs12975781 (LD-group 5) was significantly associated with “hunger” (β = −0.388, *p* = 0.023) and rs12611028 (LD-group 1) with alcohol consumption (OR = 1.625, *p* = 0.020). Furthermore, there was a significant association of rs4021 (OR = 1.292, *p* = 0.038; LD-group 3) and rs838136 (OR = 1.299, *p* = 0.030; LD-group 8) with smoking status (Table 10 and Supplementary File 7).

## 4. Discussion

### 4.1. Association of FGF21 with Food and Drug Craving

Genetic variants within or near the *FGF21* locus have been shown to be associated with eating behavior, consumption of addictive substances and metabolic and anthropometric parameters [31,32,33,34,35]. FGF21 administration in mice suppresses alcohol and sweets consumption, accompanied by a lower concentration of dopamine in the central nervous system implicating its potential role in addictive behavior [26]. In the present work, we investigated the association of serum FGF21 with food and drug craving in humans.

The present study revealed a significant negative association of serum FGF21 concentrations with the eating behavior domain of disinhibition while no significant associations with the domains of hunger or restraint were found. It is of note, that Löffler et al. defined an additional factor based on the Three-Factor-Eating-Questionnaire (German version: FEV) named ‘uncontrolled eating’ which consists of items of the domains of ‘disinhibition’ and ‘hunger’ to show that this factor conveys the strongest positive correlation with BMI [42]. It has to be taken into account that despite the robust evidence for the relationships between FGF21 and BMI as well as disinhibition, the current knowledge does not allow drawing any conclusions with regard to possible mechanistic pathways. Nevertheless, FGF21 is known to be elevated in patients with T2D, obesity and with MS [10,11,21], mostly having a higher BMI. Since FGF21 induces weight loss in obese mice [15] and in diabetic monkeys [51] as well as FGF21 analogues do in humans [27,28], it might be assumed that elevated FGF21 levels in humans may reflect a similar pattern. Whether the effect of FGF21 on obesity is mediated by eating behavior, or changes in FGF21 and the disinhibition domain are driven by obesity (BMI) itself, or whether FGF21 is a confounder of the association between disinhibition and BMI will need to be clarified in further studies focusing on mechanistic pathways underlying changes in FGF21 concentrations. It should also be taken into account, that findings in mice suggested development of FGF-21 resistance in obesity [52].

In the present study, we highlight the role of FGF21 in alcohol consumption. Søberg et al. showed elevated serum FGF21 levels in humans after acute alcohol consumption and a higher basal rate of FGF21 after three days of higher alcohol consumption on events like the German “Oktoberfest” [8]. Studies in mice revealed that administration of FGF21 reduced alcohol intake [8,26]. Further studies conducted in mice provide evidence for a liver-brain axis of FGF21 regulating alcohol intake via β-Klotho [53] and suggest that FGF21 protects the liver from alcohol associated injuries [54]. Taken together, it might be hypothesized that an endogenous increase of FGF21 after alcohol consumption, either acute or chronic, could be part of a negative feedback mechanism to reduce further alcohol consumption.

Finally, we found a significant positive association of serum FGF21 with smoking, and a negative association with coffee consumption. In contrast to our findings a previously conducted study in smokers revealed no significant effect of smoking stop on FGF21 serum concentrations [55] and the existing literature regarding the association of genetic variation in *FGF21* and smoking is inconsistent. In a study conducted by Frayling et al. only a trend was found towards an association of the rs838133 A-allele with being former smoker, whereas no effect at all was observed with being current smoker [31]. In contrast, Søberg et al. showed a significant association of the rs838133 A-allele with daily smoking and a trend towards association with occasional smoking [32].

Similarly, existing literature concerning the association between genetic variation in *FGF21* and coffee consumption is controversial. Whereas Søberg et al., did not find a significant association of the rs838133 A-allele with coffee consumption [32] which is in line with our present findings, Frayling et al., reported a significant association of the rs838133 A-allele with lower risk of being a coffee drinker and with reduced number of cups of coffee consumed per day [31].

### 4.2. Association of FGF21 with Adipokines

In the present study we observed that circulating serum FGF21 was significantly correlated with AFABP and chemerin, but also with progranulin and PENK independent of sex, age and BMI. It has to be acknowledged that a correlation of FGF21 with adiponectin seemed to be independent of age, but not sex and BMI (data not shown).

FGF21 is a suppressor of IGF-1 secretion in vitro [21] as well as of growth hormone activity by either decreasing expression of IGF-1 or upregulating its binding protein or the suppressor of cytokine signaling 2 (SOCS2) [22]. Consequently, serum FGF21 concentrations correlated inversely with serum IGF-1 concentrations in humans [21]. Additionally, we have recently shown that certain features of the MS are highly associated with circulating adiponectin, AFABP, chemerin and FGF21 [40]. However, it remains unclear, whether the MS may act as a driver or is simply a consequence of changes in adipokine regulation. Moreover, accumulating evidence points to a direct interaction between individual adipokines. For example, a study in mice conducted by Lin et al. showed that the adiponectin secreted by adipocytes is required for FGF21 to exert its effects on energy metabolism and insulin sensitivity in tissues like liver or skeletal muscle [56]. Another study in mice showed that FGF21 increases adiponectin secretion [24]. It is noteworthy that adiponectin itself plays an important role in mediating insulin sensitivity [57] and is associated with lower risk of T2D [58]. Adiponectin, as it correlates inversely with TG, insulin resistance, C-reactive protein and waist circumference and positively with HDL-C [40,59], is when decreased a potential marker of the MS [60]. A study in Chinese youths demonstrated that obesity is associated with higher FGF21 on the one hand and higher and lower adiponectin levels on the other hand [23], which is also in line with our findings. This point is of relevance when considering a possible FGF21 resistance or insulin resistance in obesity and T2D.

### 4.3. Effects of Genetic Variation within the FGF21 Locus

Based on previously published associations and pharmacological effects of FGF21, including genetic association studies mainly focusing on rs838133, rs838145 and rs838147 [31,32,33,34], we investigated the association of *FGF21* genetic variants (spanning the region of *FGF21* ±10kb) with FGF21 serum concentrations, *FGF21* expression in PBMCs, metabolic traits, adipokines, as well as food and drug craving. Significant associations were mainly seen with LDL-C with 9 SNPs from 4 LD-clusters, thus being in line with previously published studies reporting genetic association of *FGF21* [49] and association of serum FGF21 with LDL-C [12]. Based on the present analyses we identified three variants being associated with FGF21 serum concentrations as well as variants associated with the eating behavior trait ‘hunger’, alcohol consumption and smoking status. Interestingly, genetic variants in the co-receptor of FGF21, β-Klotho, through which FGF21 regulates alcohol intake, have previously been shown to be associated with alcohol consumption [53,61].

Chu et al. and Frayling et al. have previously shown that the rs838133 A-allele is significantly associated with lower protein and fat intake but higher carbohydrate intake in large-scale genome-wide association studies (GWAS) [31,33]. Furthermore, Frayling et al. reported the minor A-allele to be associated with higher alcohol intake, waist-to-hip ratio (WHR), blood pressure, LDL-C, γGT, TG, body fat, AP and coffee consumption [31,49,50]. Søberg et al. found the rs838133 A-allele to be associated with higher candy snacking and nominally associated with higher alcohol consumption and daily smoking [32]. In contrast to the study of Frayling et al. [31], no association of genetic variation with coffee consumption was found [32]. In the present study, a significant association was replicated only between rs838133 and LDL-C. In line with previous findings [49], the A-allele was associated with higher LDL-C in our study as well. Furthermore, we could replicate a trend towards an association of rs838133 with disinhibition (*p* = 0.053) and a trend with AP (*p* = 0.077). Diverging results in the present study may be a consequence of the smaller sample size and thereby limited statistical power. Beyond that, different questionnaires were applied to assess alcohol and coffee consumption, eating behavior and smoking habits. Previous genetic studies did not find an association of *FGF21* genetic variants and corresponding LD-partners with *FGF21* expression in liver [31] or blood [35,62].

Also, we could not find an association of genetic variants with *FGF21* expression in PBMCs. However, the present findings show a trend toward an association between serum FGF21 levels and its expression in PBMCs.

In the context of genetic variation, two genetic variants which map outside the here examined *FGF21* locus have to be mentioned: rs838145 and rs838147 [32,34,35]. Both variants are in LD (*R*^2^ 0.8) with rs838133. Rs838133 and rs838145 are known to be associated with higher sugar intake and sweet snacking [32]. Furthermore the rs838145 G-allele or the corresponding alleles of their LD-partner were found to be associated with higher carbohydrate, lower fat intake higher serum concentrations of FGF21 but not with mRNA amount in blood [35,62,63]. Consistently, the A-allele of rs838145 was reported to be associated with higher fat intake [63] and lower alcohol consumption [64]. The rs838147 alleles were found to interact with differences in dietary macronutrient intake and the subjects’ preference for it [34] and the A-allele was found to be significantly associated with higher carbohydrate intake on genome-wide scale [35]. However, we could not replicate any of these associations in our analysis. This may be attributed to the fact that we used the FEV questionnaires instead of measuring macronutrient intake. Furthermore, as mentioned above, the sample sizes were different between the studies, strongly influencing the statistical power to detect effects of genetic variants on measured phenotypes. However, they all showed a significant effect on LDL-C, with rs838145 conveying the strongest association (β = 0.040, *p* 0.001).

## 5. Conclusions

In summary, our study provides novel findings that may promote further work towards better understanding of the role of FGF21 in regulation of behavioral and metabolic traits related to obesity. Nevertheless, the interpretation of the present results is limited as no correction for multiple testing was applied and therefore, validation in large-scale association studies is warranted. However, our study clearly suggests a link between FGF21 and eating behavior, alcohol consumption, smoking and coffee consumption, which may comprise a component of food and drug craving. Whether lower dopamine concentration in the central nervous system are implicated in these associations like it has been reported in mice [26] remains to be elucidated in humans. Furthermore, we present new results regarding the relationship between FGF21 and other adipocytokines that may uncover and help to understand complex pathways in the pathophysiology of metabolic diseases. Finally, the role of FGF21 in the pathophysiology of metabolic diseases is supported by our genetic analyses providing evidence for a link between genetic variation within *FGF21* and metabolic traits, in particular those related to lipid metabolism.

## Figures and Tables

**Table 1:** Baseline characteristics of the Sorbs cohort.

	Total	Males (Above the Limit of Detection)	Females (Above the Limit of Detection)	*p* (Males vs. Females)
* n *	1046 (419 m/620 f)	330	450	
**Anthropometric traits**				
Age (years)	44 (25)	50 (23)	51 (24)	0.313
BMI (kg/m^2^)	26.0 (5.6)	27.0 (4.6)	26.9 (7.8)	0.287
WHR	0.88 (0.17)	0.96 (0.12)	0.83 (0.11)	**2.599 × 10^−60^**
Height (cm)	172 (13)	177 (10)	164 (9)	**7.103 × 10^−87^**
Bodyweight (kg)	75.0 (20.0)	84.5 (16.0)	71.0 (17.0)	**3.004 × 10^−36^**
SBP (mmHg)	134.25 (25.00)	142.50 (21.00)	133.25 (25.00)	**1.801 × 10^−11^**
**Glucose/insulin traits**				
T2D (yes/no)	114/921	41/286	61/389	0.678
FG (mmol/L)	5.29 (0.76)	5.54 (0.69)	5.28 (0.76)	**3.608 × 10^−10^**
FI (pmol/L)	33.90 (28.10)	33.75 (28.60)	37.20 (30.70)	**0.038**
HbA1c (%)	5.3 (0.5)	5.4 (0.6)	5.4 (0.6)	0.989
HOMA-IR	1.13 (1.41)	1.45 (1.38)	1.49 (1.48)	0.561
**Lipid traits**				
LLM (yes/no)	120/919	46/284	56/394	0.541
TG (mmol/L)	1.06 (0.75)	1.35 (0.96)	1.02 (0.68)	**4.830 × 10^−11^**
LDL-C (mmol/L)	3.34 (1.26)	3.57 (1.22)	3.32 (1.27)	**0.002**
HDL-C (mmol/L)	1.59 (0.58)	1.42 (0.45)	1.7 (0.56)	**3.532 × 10^−21^**
**Liver metabolism**				
γGT (µlat/L)	0.38 (0.44)	0.50 (0.54)	0.26 (0.20)	**9.515 × 10^−36^**
ALAT (µkat/L)	0.41 (0.27)	0.49 (0.28)	0.32 (0.17)	**4.911 × 10^−40^**
ASAT (µkat/L)	0.45 (0.16)	0.50 (0.17)	0.41 (0.12)	**7.342 × 10^−29^**
AP (µkat/L)	0.99 (0.41)	1.13 (0.36)	1.03 (0.47)	**1.400 × 10^−5^**
**Adipokines**				
FGF21 (ng/L)	84.48 (162.88)	101.31 (147.90)	92.39 (113.66)	**0.023**
Irisin (µg/mL)	0.76 (0.39)	0.76 (0.36)	0.81 (0.38)	**0.018**
Adiponectin(µg/mL)	14.79 (8.12)	13.68 (6.21)	17.40 (6.99)	**2.730 × 10^−17^**
AFABP4 (µg/L)	12.64 (15.38)	13.76 (11.57)	24.53 (22.36)	**1.663 × 10^−25^**
Chemerin (ng/mL)	113.38 (44.70)	118.12 (47.00)	124.69 (55.60)	**0.001**
Vaspin (ng/mL)	0.52 (1.11)	0.36 (0.41)	0.59 (0.97)	**4.501 × 10^−18^**
Progranulin (ng/mL)	106.50 (37.47)	109.55 (36.36)	110.15 (35.03)	0.430
AGF (µg/L)	37.78 (35.36)	39.69 (38.48)	37.78 (40.10)	0.760
IGF-1 (ng/mL)	169.20 (77.45)	156.05 (74.85)	150.05 (76.90)	**0.023**
pro-NT (pmol/L)	113.97 (62.54)	109.47 (56.13)	116.53 (61.91)	0.113
PENK (pmol/L)	57.75 (20.12)	55.10 (18.69)	57.79 (22.23)	**0.010**
**Luxury food**				
Smoker(yes/no)	351/688	180/150	92/358	**5.436 × 10^−23^**
cigarette (packs/year)	5.00 (8.50)	8.13 (17.00)	2.35 (4.18)	**8.838 × 10^−14^**
Score alcohol	3 (3)	4 (2)	2 (1)	**2.690 × 10^−49^**
Score coffee	1 (1)	2 (1)	2 (1)	**0.911**
**Eating behavior**				
Score disinhibition	4 (4)	3 (3)	4 (5)	**0.003**
Score hunger	3 (4)	3 (4)	4 (4)	**0.014**
Score restraint	7.0 (7.5)	6.0 (5.5)	9.0 (8.0)	**1.871 × 10^−9^**

Data were shown as median (interquartile range) or total number; TG, LDL-C, HDL-C were shown for subjects without lipid lowering medication. Assessment of group-differences were calculated by Mann-Whitney-U test (continuous parameters) or Chi-square-test (discrete variables: T2D and smokers). *p*-values presented comparison of males vs. females including only subjects with detectable FGF21 serum levels. A *p*-value 0.05 was considered to be significant and was highlighted in bold. *n* = number of subjects; m = males; f = females; *p* = *p*-value; BMI = body-mass index; WHR = waist-to-hip ratio; SBP = systolic blood pressure; T2D = type 2 diabetes; FG = fasting glucose; FI = fasting insulin; HbA1c = hemoglobin A1c, long term glucose level; HOMA-IR = homeostasis assessment model of insulin resistance; LLM = lipid lowering medication; TG = triglyceride; LDL-C = low density lipoprotein cholesterol; HDL-C = high density lipoprotein cholesterol; γGT = gamma-glutamyltransferase; ALAT = alanine aminotransferase; ASAT = aspartate aminotransferase; AP = alkaline phosphatase; FGF21 = fibroblast growth factor-21; AFABP = adipocyte fatty acid binding protein; AGF = angiopoietin-related growth factor; IGF-1 = insulin like growth factor 1; pro-NT = pro-neurotensin; PENK = pro-enkephalin.

**Table 2:** Baseline characteristics of the validation cohort.

	Total	Males	Females	*p*
* n *	704	387	317	
Age (years)	54 (20)	52 (22)	57 (16)	**0.004**
BMI (kg/m^2^)	37.8 (13.14)	37.8 (15.86)	37.4 (9.15)	0.155
FGF21 (ng/L)	198.84 (207.29)	184.73 (196.47)	212.98 (224.69)	**0.005**
T2D (yes/no)	341/371	156/228	180/137	**2.00 × 10** **^−^** **^5^**
Smoker (yes/no)	114/518	62/295	51/218	0.608

Continuous normally distributed data are shown as mean with standard deviation. FGF21 is shown as median with interquartile rage; for T2D and smokers, total numbers are shown. Group differences were calculated by Mann-Whitney-U test (continuous parameters) or Chi-square-test (discrete variables: T2D and smokers). *p*-values presented comparison of males vs. females. A *p*-value 0.05 was considered to be significant and was highlighted in bold. *n* = number of subjects; *p* = *p*-value; BMI = body-mass index; T2D = type 2 diabetes; FGF21 = fibroblast growth factor-21.

**Table 3:** LD-groups of the investigated *FGF21* SNPs.

LD-Group	SNP	A1	A2	MAF
1	**rs4015**	**T**	**C**	**0.110**
	rs12611028	T	C	0.095
2	**rs838133**	**G**	**A**	**0.493**
	rs838144	T	C	0.483
3	**rs4021**	**G**	**A**	**0.245**
4	**rs12975033**	**T**	**A**	**0.401**
	rs8104897	A	C	0.398
	rs8105137	A	G	0.398
	rs8106205	C	T	0.414
	rs11667321	G	A	0.414
5	**rs12975781**	**T**	**C**	**0.341**
6	**rs3826821**	**A**	**G**	**0.065**
7	**rs838143**	**A**	**G**	**0.081**
8	**rs838136**	**C**	**T**	**0.285**
9	**rs499765**	**G**	**C**	**0.331**

The representative SNP of each LD-group is indicated in bold letters. A1 presents the minor allele and A2 the major allele. LD-plot 9 does not pass the Hardy-Weinberg-Equilibrium with *p* 0.0001 and was therefore excluded from further analysis. LD = linkage disequilibrium; FGF21 = fibroblast growth factor 21; SNP = single nucleotide polymorphism; MAF = minor allele frequency.

**Table 4:** Association of FGF21 with eating behavior and food and drug craving in the Sorbs.

Dependent Variables	FGF21 (Unadjusted)		FGF21 (Adjusted for Age, Sex, BMI)		
**Linear regression**					
	**β**	*p*	β	*p*	* n *
Disinhibition	**−0.152**	**0.001**	**−0.158**	**2.170 × 10^−4^**	465
Hunger	−0.061	0.184	−0.052	0.260	465
Restraint	0.066	0.153	0.045	0.318	465
Packyears	**0.239**	**6.800 × 10^−5^**	**0.111**	**0.030**	268
**Generalized linear model**					
	OR	*p*	OR	*p*	* n *
Alcohol consumption	**1.315**	**1.300 × 10^−5^**	**1.281**	**1.510 × 10^−4^**	768
Coffee consumption	**0.872**	**0.029**	**0.800**	**0.001**	761
**Binary logistic regression**					
	OR	*p*	OR	*p*	* n *
Smoker (yes/no)	**1.341**	**7.200 × 10^−5^**	**1.394**	**6.700 × 10^−5^**	780

Prior to statistical analysis the variables packyears, serum FGF21, BMI and age were ln-transformed. Assessments were done using linear regression for eating behavior and packyears, generalized linear model for alcohol and coffee consumption and binary logistic regression for smoking status. A *p*-value 0.05 was considered to be significant and was highlighted in bold. FGF21 = fibroblast growth factor 21; BMI = body-mass-index; OR = odds ratio; β = regression coefficient; *p* = *p*-value; *n* = number of subjects.

**Table 5:** FGF21 and alcohol consumption and smoking in the validation cohort.

Dependent Variables	FGF21 (Unadjusted)		FGF21 (Adjusted for Age, Sex, BMI)		
**Linear regression**					
	**β**	*p*	β	*p*	* n *
Alcohol consumption	**0.162**	**3.00 × 10** **^−^** **^4^**	**0.164**	**1.00 × 10** **^−^** **^3^**	492
**Binary logistic regression**					
	OR	*p*	OR	*p*	* n *
Smoker (yes/no)	**1.322**	**0.011**	**1.666**	**4.10 × 10** **^−^** **^5^**	614

Prior to analysis, the variables serum FGF21, BMI and age were ln-transformed. Alcohol consumption was operationalized by the scale ‘food alcoholic’. Assessments were done using linear regression for alcohol consumption and binary logistic regression for smoking status. A *p*-value 0.05 was considered to be significant and was highlighted in bold. FGF21 = fibroblast growth factor 21, BMI = body-mass-index; OR = odds ratio; β = regression coefficient; *p* = *p*-value; *n* = number of subjects.

**Table 6:** Correlation analyses of adipokines and serum FGF21 in the Sorbs cohort.

Adipokine	* n *	Spearman’s Rank Correlation		Partial Correlation (Adjusted for Age, Sex, BMI)	
		Spearman’s Rank Coefficient	*p*	Correlation Coefficient	*p*
Irisin	777	−0.048	0.178	−0.060	0.150
Adiponectin	725	**−0.092**	**0.013**	−0.068	0.105
AFABP	780	**0.271**	**1.393×10^−14^**	**0.248**	**2.444×10^−10^**
Chemerin	779	**0.246**	**3.294×10^−12^**	**0.200**	**5.586×10^−9^**
Vaspin	779	0.029	0.424	0.067	0.109
Progranulin	780	**0.123**	**0.001**	**0.098**	**0.019**
AGF	628	0.027	0.498	−0.042	0.322
IGF-1	780	**−0.256**	**4.195×10^−13^**	**−0.195**	**3.000×10^−6^**
pro-NT	777	0.020	0.584	0.021	0.614
PENK	777	0.021	0.559	**0.092**	**0.028**

Prior statistical analysis, continuous variables were ln-transformed. Assessments were done using Spearman’s rank correlation and partial correlation with adjustments for sex, age and BMI. A *p*-value 0.05 was considered to be significant and was highlighted in bold. *n* = number of subjects; *p* = *p*-value; BMI = body-mass-index; FGF21 = fibroblast growth factor 21; AFABP = adipocyte fatty-acid-binding protein; AGF = angiopoietin-related growth factor; IGF-1 = insulin-like growth factor 1; pro-NT = pro-neurotensin; PENK = pro-enkephalin.

**Table 7:** Association of representative SNPs for each LD-group with glucose and lipid metabolism, and liver parameters in the Sorbs cohort.

		LD 1	LD 2	LD 3	LD 4	LD 5	LD 6	LD 7	LD 8	* n *
Fasting glucose	β	−0.018	0.005	−0.003	0.003	0.004	0.016	0.012	0.002	
	s.e.	0.013	0.008	0.010	0.008	0.008	0.016	0.015	0.009	946
	*p*	0.183	0.486	0.777	0.746	0.624	0.315	0.424	0.857	
Fasting insulin	β	**0.128**	0.030	0.049	0.018	0.030	**0.111**	0.044	0.026	
	s.e.	**0.042**	0.025	0.030	0.025	0.027	**0.052**	0.046	0.029	946
	*p*	**0.002**	0.214	0.104	0.468	0.269	**0.033**	0.349	0.371	
HOMA-IR	β	**0.109**	0.047	0.044	0.030	0.036	**0.135**	0.066	0.046	
	s.e.	**0.046**	0.027	0.033	0.0274	0.029	**0.056**	0.051	0.032	935
	*p*	**0.019**	0.079	0.180	0.274	0.215	**0.017**	0.196	0.155	
HbA1c	β	−0.005	0.001	0.001	0.001	0.001	0.002	0.001	0.008	
	s.e.	0.007	0.004	0.005	0.004	0.004	0.009	0.008	0.005	953
	*p*	0.472	0.884	0.834	0.819	0.804	0.845	0.859	0.086	
LDL-C	β	−0.031	−**0.034**	−0.021	−**0.044**	−**0.032**	0.016	0.021	−**0.041**	
	s.e.	0.022	**0.012**	0.015	**0.012**	**0.013**	0.025	0.023	**0.015**	845
	P	0.150	**0.005**	0.165	**4.482** × **10^−4^**	**0.018**	0.538	0.352	**0.005**	
HDL-C	β	0.010	−0.006	−0.001	−0.017	−0.016	−0.028	0.021	0.010	
	s.e.	0.017	0.010	0.012	0.010	0.011	0.021	0.019	0.012	845
	*p*	0.578	0.511	0.962	0.100	0.129	0.178	0.263	0.417	
TG	β	0.043	−0.031	0.017	−0.030	−0.018	0.072	0.008	−0.012	
	s.e.	0.038	0.021	0.026	0.022	0.023	0.045	0.040	0.026	845
	*p*	0.260	0.142	0.517	0.169	0.427	0.107	0.833	0.642	
γGT	β	0.052	-0.035	−0.027	−0.001	−0.007	0.072	−**0.113**	−0.043	
	s.e.	0.048	0.028	0.035	0.029	0.030	0.059	**0.053**	0.034	958
	*p*	0.287	0.205	0.429	0.965	0.828	0.224	**0.034**	0.198	
AP	β	0.014	0.022	0.009	0.020	0.014	0.020	0.003	0.018	
	s.e.	0.021	0.012	0.015	0.012	0.013	0.026	0.023	0.015	958
	*p*	0.521	0.077	0.530	0.115	0.304	0.439	0.912	0.210	
ALAT	β	0.006	−0.026	−0.017	−0.025	−0.023	0.015	−0.012	−0.014	
	s.e.	0.030	0.017	0.021	0.017	0.019	0.036	0.033	0.021	958
	*p*	0.850	0.133	0.425	0.145	0.217	0.685	0.724	0.508	
ASAT	β	0.020	−0.020	−0.006	−**0.022**	−0.013	0.007	−0.003	−0.016	
	s.e.	0.019	0.011	0.013	**0.011**	0.012	0.023	0.021	0.013	958
	*p*	0.281	0.068	0.661	**0.047**	0.263	0.754	0.865	0.231	

The presented associations were given for the representative SNP within each LD-group. Prior to analysis, all variables were ln-transformed except the covariate sex. *p*-values were adjusted for: sex, age, BMI and a *p*-value 0.05 was considered to be significant and was highlighted in bold. Linear regression including all subjects was conducted. For lipid parameters only subjects without lipid lowering medication were analyzed. LD = linkage disequilibrium; HOMA-IR = homeostasis assessment model of insulin resistance; HbA1c = hemoglobin A1c; LDL-C = low density lipoprotein cholesterol; HDL-C = high density lipoprotein cholesterol; TG = triglyceride; γGT = gamma-glutamyltransferase; AP = alkaline phosphatase; ALAT = alanine aminotransferase; ASAT = aspartate aminotransferase; BMI = body-mass index; *p* = *p*-value; β = regression coefficient; s.e. = standard error; *n* = number of subjects.

**Table 8:** Association of representative SNPs for each LD-group with FGF21-parameters and anthropometric parameters in the Sorbs cohort.

		LD 1	LD 2	LD 3	LD 4	LD 5	LD 6	LD 7	LD 8	*n*
FGF21- serum level	β	**0.227**	0.068	0.048	0.081	0.076	−0.155	0.001	0.079	
	s.e.	**0.088**	0.051	0.063	0.052	0.056	0.108	0.098	0.061	724
	*p*	**0.010**	0.180	0.453	0.121	0.173	0.150	0.989	0.195	
*FGF21*- expression	β	−0.001	1.261 × 10^−4^	2.669 × 10^−4^	−0.001	−0.001	−2.052 × 10^−4^	0.003	0.001	
	s.e.	0.002	0.001	0.001	0.001	0.001	0.002	0.002	0.001	938
	*p*	0.424	0.900	0.830	0.313	0.357	0.923	0.165	0.571	
BMI	β	−0.010	−0.006	−0.007	−0.005	−0.005	0.004	−0.002	−0.001	
	s.e.	0.012	0.007	0.008	0.007	0.007	0.014	0.013	0.008	958
	*p*	0.430	0.358	0.390	0.517	0.523	0.785	0.901	0.940	
WHR	β	0.005	3.225 × 10^−4^	0.004	0.001	0.002	4.664 × 10^−4^	4.915 × 10^−4^	0.003	
	s.e.	0.005	0.003	0.004	0.003	0.003	0.006	0.006	0.003	957
	*p*	0.283	0.912	0.315	0.724	0.622	0.940	0.929	0.403	
Height	β	−0.002	−0.002	−**0.004**	0.001	0.001	0.004	−**0.009**	−0.003	
	s.e.	0.003	0.002	**0.002**	0.002	0.002	0.003	**0.003**	0.002	958
	*p*	0.515	0.276	**0.031**	0.726	0.418	0.228	**0.003**	0.069	
Weight	β	−0.005	−0.004	−**0.009**	8.199 × 10^−5^	0.002	0.008	−**0.018**	−0.007	
	s.e	0.005	0.003	**0.004**	0.003	0.003	0.007	**0.006**	0.004	958
	*p*	0.323	0.160	**0.022**	0.980	0.647	0.226	**0.003**	0.051	
SBP	β	−0.002	−0.002	−0.008	−0.057	0.004	0.020	−0.009	−4.078 × 10^−4^	
	s.e.	0.008	0.005	0.006	0.005	0.005	0.010	0.009	0.006	958
	*p*	0.850	0.659	0.193	0.991	0.475	0.054	0.322	0.944	

The presented associations were given for the representative SNP within each LD-group. Prior analysis, all variables were ln-transformed except the covariate sex. *p*-values were adjusted for: sex, age, BMI and *p*-values 0.05 were considered to be significant and highlighted in bold. Association analysis was done using linear regression analysis. LD = linkage disequilibrium; FGF = fibroblast growth factor; BMI = body-mass index; WHR = waist-to-hip ratio; SBP = systolic blood pressure; *p* = *p*-value; β = regression coefficient; s.e. = standard error; *n* = number of subjects.

**Table 9:** Association of representative SNPs for each LD-group with adipokines in the Sorbs cohort.

		LD 1	LD 2	LD 3	LD 4	LD 5	LD 6	LD 7	LD 8	* n *
IGF-1	β	−0.014	−0.017	0.001	−**0.029**	−0.028	0.020	0.038	0.004	
	s.e.	0.023	0.013	0.017	**0.014**	0.015	0.029	0.026	0.016	958
	*p*	0.562	0.208	0.955	**0.034**	0.057	0.491	0.144	0.783	
Chemerin	β	−0.001	−0.014	−0.017	−0.014	−0.015	0.010	−0.017	−0.021	
	s.e.	0.024	0.014	0.017	0.014	0.015	0.030	0.027	0.017	929
	*p*	0.961	0.306	0.312	0.337	0.332	0.748	0.528	0.203	
Progranulin	β	0.027	0.007	0.013	0.008	0.007	0.019	0.005	0.006	
	s.e.	0.019	0.011	0.014	0.011	0.012	0.023	0.021	0.013	957
	*p*	0.156	0.513	0.336	0.472	0.541	0.424	0.801	0.668	
AFABP	β	**0.088**	0.027	0.026	0.019	0.013	0.020	0.010	0.029	
	s.e.	**0.040**	0.023	0.029	0.024	0.025	0.050	0.045	0.028	930
	*p*	**0.028**	0.245	0.364	0.416	0.612	0.690	0.827	0.296	
Adiponectin	β	**0.057**	−0.011	0.014	−0.014	−0.008	0.014	0.008	−0.004	
	s.e.	**0.026**	0.015	0.019	0.015	0.016	0.032	0.029	0.018	928
	*p*	**0.031**	0.482	0.458	0.343	0.637	0.660	0.786	0.807	
Irisin	β	−0.001	−0.002	−0.017	0.002	−0.008	0.002	−0.023	−0.004	
	s.e.	0.025	0.014	0.018	0.015	0.016	0.031	0.027	0.017	954
	*p*	0.984	0.878	0.336	0.869	0.624	0.939	0.401	0.810	
Vaspin	β	0.046	−0.030	0.031	−0.044	−0.030	0.129	0.036	0.029	
	s.e.	0.078	0.045	0.055	0.046	0.049	0.095	0.085	0.054	952
	*p*	0.562	0.500	0.577	0.333	0.536	0.173	0.674	0.586	
AGF	β	0.053	0.065	−0,058	0.066	0.021	0.154	0.055	−0.008	
	s.e.	0.101	0.057	0.072	0.059	0.063	0.125	0.110	0.069	768
	*p*	0.600	0.256	0.999	0.263	0.737	0.218	0.616	0.904	
pro-NT	β	−0.030	−0.005	−**0.044**	−0.003	−0.011	−0.030	−0.023	−0.016	
	s.e.	0.031	0.018	**0.022**	0.018	0.019	0.037	0.034	0.021	953
	*p*	0.323	0.758	**0.045**	0.869	0.562	0.438	0.495	0.449	
PENK	β	0.015	0.001	0.003	−0.006	0.004	**0.066**	0.029	0.014	
	s.e.	0.021	0.012	0.015	0.012	0.013	**0.025**	0.023	0.014	953
	*p*	0.480	0.958	0.827	0.604	0.770	**0.009**	0.195	0.336	

The presented associations are given for the representative SNP within each LD-group. Prior to analysis, all variables were ln-transformed except the covariate sex. *p*-values were adjusted for: sex, age, BMI and a *p*-value 0.05 was considered to be significant and highlighted in bold. Linear regression including all subjects was conducted. LD = linkage disequilibrium; IGF-1 = insulin like growth factor 1; AFABP = adipocyte fatty acid-binding protein; AGF = angiopoietin-related growth factor; pro-NT = pro-neurotensin; PENK = pro-enkephalin; BMI = body-mass index; *p* = *p*-value; β = regression coefficient; s.e. = standard error; *n* = number of subjects.

**Table 10:** Association of representative SNPs for each LD-group with food and drug craving in the Sorbs cohort.

		LD 1	LD 2	LD 3	LD 4	LD 5	LD 6	LD 7	LD 8	* n *
Disinhibition	β	−0.014	−0.295	0.124	−0.228	−0.242	0.034	−0.148	0.014	
	s.e.	0.259	0.152	0.190	0.154	0.167	0.314	0.287	0.186	572
	*p*	0.957	0.053	0.514	0.141	0.148	0.915	0.608	0.942	
Hunger	β	−0.070	−0.241	−0.065	−0.251	−**0.388**	0.154	−0.027	−0.118	
	s.e.	0.263	0.155	0.193	0.157	**0.170**	0.319	0.292	0.190	572
	*p*	0.790	0.120	0.739	0.110	**0.023**	0.629	0.927	0.533	
Restraint	β	−0.554	−0.089	0.017	−0.256	−0.176	0.176	0.544	−0.178	
	s.e.	0.447	0.264	0.329	0.267	0.290	0.543	0.497	0.322	572
	*p*	0.216	0.736	0.958	0.339	0.544	0.746	0.274	0.580	
Smoking yes/no	OR	1.188	1.105	**1.292**	1.100	1.024	1.054	1.058	**1.299**	
	s.e.	0.173	0.100	**0.123**	0.103	0.110	0.210	0.190	**0.121**	958
	*p*	0.321	0.320	**0.038**	0.364	0.827	0.803	0.767	**0.030**	
Cigarettes (Packyears)	β	0.021	0.140	0.070	0.145	0.072	−0.204	0.051	0.073	
	s.e.	0.146	0.086	0.098	0.088	0.095	0.182	0.151	0.098	323
	*p*	0.883	0.105	0.476	0.102	0.450	0.262	0.734	0.455	
Coffee	OR	0.904	1.057	0.973	1.045	1.031	0.953	0.979	1.030	
	s.e.	0.160	0.092	0.114	0.094	0.101	0.194	0.175	0.111	931
	*p*	0.530	0.551	0.808	0.638	0.763	0.804	0.903	0.788	
Alcohol	OR	1.434	1.010	1.150	1.051	1.027	1.204	0.973	0.982	
	s.e.	0.198	0.114	0.144	0.117	0.126	0.238	0.230	0.142	952
	*p*	0.069	0.928	0.332	0.671	0.833	0.436	0.906	0.900	

The presented associations are given for the representative SNP within each LD-group. Prior analysis, all variables were ln-transformed except smoking, alcohol and coffee consumption, scores of eating behavior and sex. *p*-values were adjusted for: sex, age, BMI and a *p*-value 0.05 was considered to be significant and highlighted in bold. Linear regression including all subjects was conducted. The variables alcohol, coffee and smoking have been analyzed using ordinal regression and including all subjects. LD = linkage disequilibrium; OR = odds ratio; *p* = *p*-value; β = regression coefficient; s.e. = standard error; *n* = number of subjects.

## Data Availability

All data generated or analyzed during this study are included in this published article.

## Supplementary Materials

The following are available online at https://www.mdpi.com/article/10.3390/biomedicines9040345/s1. Supplementary File 1: Brief description of analyzed genetic variants in the *FGF21* locus ± 10kb. Supplementary File 2: Association of *FGF21* genetic variants with lipid traits. Supplementary File 3: Association of *FGF21* genetic variants with serum FGF21, *FGF21* gene expression and further adipocytokines. Supplementary File 4: Association of *FGF21* genetic variants with anthropometric traits. Supplementary File 5: Association of *FGF21* genetic variants with glucose traits. Supplementary File 6: Association of *FGF21* genetic variants with liver parameters. Supplementary File 7: Association of FGF21 genetic variants with eating behavior and parameters of food and drug craving.
